# Supplementary material for: Integration of bioinformatics and identification of the role of m6A genes in NAFLD
Source: PLoS One. 2025 May 28;20(5):e0321757. doi: 10.1371/journal.pone.0321757 (PMC12119021; doi:10.1371/journal.pone.0321757)
Supplement: S1 Table — (PDF) [file pone.0321757.s001.pdf]

**S1 Table. NAFLD Data Set Information List.**

| | **GSE89632** | **GSE37031** | **GSE135251** |
|---|---|---|---|
| Platform | GPL14951 | GPL14877 | GPL18573 |
| Species | Homo sapiens | Homo sapiens | Homo sapiens |
| Tissue | liver | liver | liver |
| Samples in NAFLD group | 39 | 8 | 206 |
| Samples in Control group | 24 | 7 | 10 |
| Reference | Altered hepatic gene expression in nonalcoholic fatty liver disease is associated with lower hepatic n-3 and n-6 polyunsaturated fatty acids. | Molecular interplay between Δ5/Δ6 desaturases and long-chain fatty acids in the pathogenesis of non-alcoholic steatohepatitis. | Transcriptomic profiling across the nonalcoholic fatty liver disease spectrum reveals gene signatures for steatohepatitis and fibrosis. |

GEO: Gene Expression Omnibus. NAFLD: Nonalcoholic fatty liver disease.
