# Supplementary material for: Integration of bioinformatics and identification of the role of m6A genes in NAFLD
Source: PLoS One. 2025 May 28;20(5):e0321757. doi: 10.1371/journal.pone.0321757 (PMC12119021; doi:10.1371/journal.pone.0321757)
Supplement: S2 Table — (PDF) [file pone.0321757.s002.pdf]

S2 Table. M6A-Related Genes List.

**m6A related genes list.**	
ALKBH5	YTHDC2
FTO	YTHDF1
HAKAI	YTHDF2
HNRNPA2B1	YTHDF3
HNRNPC	ZC3H13
IGF2BP1	ZCCHC4
IGF2BP2	CBLL1
IGF2BP3	TP53
KIAA1429	METTL4
METTL14	ZNF217
METTL16	EIF3A
METTL3	EIF3B
RBM15	LRPPRC
RBM15B	FMR1
VIRMA	ELAVL1
WTAP	METTL5
YTHDC1	RBMX
