# Supplementary material for: Integration of bioinformatics and identification of the role of m6A genes in NAFLD
Source: PLoS One. 2025 May 28;20(5):e0321757. doi: 10.1371/journal.pone.0321757 (PMC12119021; doi:10.1371/journal.pone.0321757)
Supplement: S3 Table — (PDF) [file pone.0321757.s003.pdf]

S3 Table. The Primer Information.

Gene	Sequence
EIF3B	FORWARD: AAGCAGCAGGCGAACACCATC
	REVERSE: CGCTAAGGCACCGTTCATACTCC
RBM15	FORWARD: CTCCGACGACCCGCAACAATG
	REVERSE: CCTAACTTCTTGCTCCGCTCACC
YTHDC1	FORWARD: ATCATCTTCCGTTCGTGCTGTCC
	REVERSE: ACACCCTTCGCTTTGGCAAGAG
WTAP	FORWARD: AGGGCAACACAACCGAAGATGAC
	REVERSE: ACCACTACCTCCTCTGCCAGTTC
IGF2BP2	FORWARD: TGTTGGTGCCATCATCGGAAAGG
	REVERSE: GCATGGATGGTGACAGGCTTCTC
GAPDH	FORWARD: ACGGCAAATTCAACGGCACAG
	REVERSE: ACACCAGTAGACTCCACGACATAC
