# Supplementary material for: Integration of bioinformatics and identification of the role of m6A genes in NAFLD
Source: PLoS One. 2025 May 28;20(5):e0321757. doi: 10.1371/journal.pone.0321757 (PMC12119021; doi:10.1371/journal.pone.0321757)
Supplement: S4 Table — (PDF) [file pone.0321757.s004.pdf]

S4 Table. GO Enrichment Analysis Results.

Ontology	ID	Description	GeneRatio	BgRatio	pvalue	p.adjust	qvalue
BP	GO:0000381	regulation of alternative mRNA splicing, via spliceosome	3/9	60/18800	2.5608E-06	0.000237731	0.000109596
BP	GO:0000380	alternative mRNA splicing, via spliceosome	3/9	77/18800	5.45178E-06	0.000237731	0.000109596
BP	GO:0001510	RNA methylation	3/9	83/18800	6.83793E-06	0.000237731	0.000109596
BP	GO:1903311	regulation of mRNA metabolic process	4/9	294/18800	6.94105E-06	0.000237731	0.000109596
BP	GO:0075522	IRES-dependent viral translational initiation	2/9	11/18800	1.11798E-05	0.000299083	0.000137879
CC	GO:0005852	eukaryotic translation initiation factor 3 complex	2/9	15/19594	1.96315E-05	0.000108625	4.81439E-05
CC	GO:0033290	eukaryotic 48S preinitiation complex	2/9	15/19594	1.96315E-05	0.000108625	4.81439E-05

CC	GO:0016607	nuclear speck	4/9	411/19594	2.21144E-05	0.000108625	4.81439E-05
CC	GO:0016282	eukaryotic 43S preinitiation complex	2/9	17/19594	2.54153E-05	0.000108625	4.81439E-05
CC	GO:0070993	translation preinitiation complex	2/9	18/19594	2.85854E-05	0.000108625	4.81439E-05
MF	GO:0003743	translation initiation factor activity	2/9	51/18410	0.000267524	0.006420565	0.003097641
MF	GO:0008135	translation factor activity, RNA binding	2/9	84/18410	0.000725343	0.0064806	0.003126605
MF	GO:0045182	translation regulator activity	3/9	406/18410	0.000810075	0.0064806	0.003126605
MF	GO:0090079	translation regulator activity, nucleic acid binding	2/9	108/18410	0.001194947	0.007169681	0.003459057
MF	GO:1904047	S-adenosyl-L-methionine binding	1/9	18/18410	0.008767127	0.038366685	0.018510243

GO: Gene Ontology; BP: biological process; CC: cellular component; MF: molecular functio
