# Supplementary material for: Integration of bioinformatics and identification of the role of m6A genes in NAFLD
Source: PLoS One. 2025 May 28;20(5):e0321757. doi: 10.1371/journal.pone.0321757 (PMC12119021; doi:10.1371/journal.pone.0321757)
Supplement: S5 Table — (PDF) [file pone.0321757.s005.pdf]

S5 Table. mRNA-miRNA Interaction Network Nodes.

miRNA	mRNA	miRNA	mRNA
hsa-miR-124-3p	EIF3B	hsa-miR-1270	IGF2BP2
hsa-let-7a-5p	IGF2BP2	hsa-miR-3064-5p	IGF2BP2
hsa-let-7b-5p	IGF2BP2	hsa-miR-23a-3p	WTAP
hsa-let-7c-5p	IGF2BP2	hsa-miR-129-5p	WTAP
hsa-let-7d-5p	IGF2BP2	hsa-miR-212-3p	WTAP
hsa-let-7e-5p	IGF2BP2	hsa-miR-23b-3p	WTAP
hsa-let-7f-5p	IGF2BP2	hsa-miR-132-3p	WTAP
hsa-miR-18a-5p	IGF2BP2	hsa-miR-455-5p	WTAP
hsa-miR-98-5p	IGF2BP2	hsa-miR-425-5p	WTAP
hsa-miR-181a-5p	IGF2BP2	hsa-miR-15a-5p	YTHDC1
hsa-miR-181b-5p	IGF2BP2	hsa-miR-16-5p	YTHDC1
hsa-miR-181c-5p	IGF2BP2	hsa-miR-103a-3p	YTHDC1
hsa-miR-221-3p	IGF2BP2	hsa-miR-107	YTHDC1
hsa-miR-222-3p	IGF2BP2	hsa-miR-15b-5p	YTHDC1
hsa-let-7g-5p	IGF2BP2	hsa-miR-137	YTHDC1
hsa-let-7i-5p	IGF2BP2	hsa-miR-195-5p	YTHDC1
hsa-miR-141-3p	IGF2BP2	hsa-miR-424-5p	YTHDC1
hsa-miR-9-5p	IGF2BP2	hsa-miR-485-5p	YTHDC1
hsa-miR-200a-3p	IGF2BP2	hsa-miR-497-5p	YTHDC1
hsa-miR-181d-5p	IGF2BP2	hsa-miR-519d-3p	YTHDC1

hsa-miR-877-5p	IGF2BP2	hsa-miR-543	YTHDC1
hsa-miR-216b-5p	IGF2BP2		

“mRNA”and“miRNA”represent node; “-”represent edge
