# Supplementary material for: Integration of bioinformatics and identification of the role of m6A genes in NAFLD
Source: PLoS One. 2025 May 28;20(5):e0321757. doi: 10.1371/journal.pone.0321757 (PMC12119021; doi:10.1371/journal.pone.0321757)
Supplement: S6 Table — (PDF) [file pone.0321757.s006.pdf]

S6 Table. mRNA-RBP Interaction Network Nodes.

mRNA	RBP	mRNA	RBP
EIF3B	ALYREF	IGF2BP2	U2AF1
EIF3B	DDX3X	IGF2BP2	U2AF2
EIF3B	ELAVL1	IGF2BP2	YTHDF1
EIF3B	FUS	RBM15	ALYREF
EIF3B	G3BP1	RBM15	ELAVL3
EIF3B	HNRNPC	RBM15	G3BP1
EIF3B	LIN28B	RBM15	HNRNPC
EIF3B	RBFOX2	RBM15	RBMX
EIF3B	RBMX	RBM15	TARDBP
EIF3B	RNPS1	RBM15	YTHDF1
EIF3B	TARDBP	RBM15	YTHDF2
EIF3B	U2AF1	WTAP	ALYREF
EIF3B	U2AF2	WTAP	CSTF2T
EIF3B	UPF1	WTAP	DDX3X
EIF3B	YTHDC1	WTAP	ELAVL1
EIF3B	YTHDF1	WTAP	FUS
IGF2BP2	ALYREF	WTAP	HNRNPC
IGF2BP2	ELAVL1	WTAP	PTBP1
IGF2BP2	G3BP1	WTAP	RBMX
IGF2BP2	HNRNPC	WTAP	TARDBP

IGF2BP2	HNRNPM	WTAP	U2AF1
IGF2BP2	IGF2BP3	WTAP	U2AF2
IGF2BP2	LIN28B	WTAP	YTHDF1
IGF2BP2	NXF1	YTHDC1	ALYREF
IGF2BP2	PTBP1	YTHDC1	ELAVL1
IGF2BP2	RBFOX2	YTHDC1	RBMX
IGF2BP2	RBMX	YTHDC1	TARDBP
IGF2BP2	RNPS1	YTHDC1	U2AF2
IGF2BP2	TARDBP	YTHDC1	YTHDC1

“mRNA”and“RBP”represent node, “-”represent edge. RBP: RNA binding protein.
