# Supplementary material for: Integration of bioinformatics and identification of the role of m6A genes in NAFLD
Source: PLoS One. 2025 May 28;20(5):e0321757. doi: 10.1371/journal.pone.0321757 (PMC12119021; doi:10.1371/journal.pone.0321757)
Supplement: S7 Table — (PDF) [file pone.0321757.s007.pdf]

S7 Table. mRNA-TF Interaction Network Nodes.

mRNA	TF	mRNA	TF
RBM15	CTCF	RBM15	CTCF
RBM15	E2F6	RBM15	CTCFL
RBM15	EGR1	RBM15	E2F1
RBM15	ATF3	RBM15	E2F4
RBM15	MAX	RBM15	EGR1
RBM15	MITF	RBM15	ELF1
RBM15	MNT	RBM15	ESR1
RBM15	MXI1	RBM15	HNF4A
RBM15	MYC	RBM15	HOXB13
RBM15	MYCN	RBM15	KMT2A
RBM15	NRF1	RBM15	MAX
RBM15	POLR2A	RBM15	MED1
RBM15	TBP	RBM15	MITF
RBM15	USF1	RBM15	MXI1
RBM15	USF2	RBM15	BHLHE40
RBM15	WDR5	RBM15	MYC
RBM15	YY1	RBM15	NFYA
RBM15	CDK9	RBM15	NOTCH1
IGF2BP2	SMARCA4	RBM15	BRD2
IGF2BP2	SUZ12	RBM15	BRD4

IGF2BP2	TFAP2A	RBM15	POLR2A
IGF2BP2	USF1	RBM15	RAD21
IGF2BP2	CEBPA	RBM15	RUNX1
IGF2BP2	CEBPB	RBM15	SMC3
IGF2BP2	EGR1	RBM15	SP1
IGF2BP2	ETV1	RBM15	SP2
IGF2BP2	FOSL2	RBM15	SUPT5H
IGF2BP2	FOXA1	RBM15	TBP
IGF2BP2	FOXA2	RBM15	TFAP2A
IGF2BP2	GABPA	RBM15	USF1
IGF2BP2	GATA6	RBM15	USF2
IGF2BP2	HNF4A	RBM15	YY1
IGF2BP2	NRF1	RBM15	ZEB1
RBM15	CREB1	RBM15	ZNF143

“mRNA”and“TF”represent node; “-”represent edge; TF: Transcription factors.
