# Supplementary material for: Integration of bioinformatics and identification of the role of m6A genes in NAFLD
Source: PLoS One. 2025 May 28;20(5):e0321757. doi: 10.1371/journal.pone.0321757 (PMC12119021; doi:10.1371/journal.pone.0321757)
Supplement: S8 Table — (PDF) [file pone.0321757.s008.pdf]

S8 Table. mRNA-Drung Interaction Network Nodes.

mRNA	drung	mRNA	drung
IGF2BP2	Silicon Dioxide	IGF2BP2	Nanotubes, Carbon
YTHDC1	bisphenol A	IGF2BP2	Nicotine
RBM15	1,2-Dimethylhydrazine	IGF2BP2	N-Methyl-3,4-methylenedioxyamphetamine
RBM15	2,2',4,4'-tetrabromodiphenyl ether	IGF2BP2	Oxygen
RBM15	Acetaminophen	IGF2BP2	Particulate Matter
RBM15	Carbon Tetrachloride	IGF2BP2	pirinixic acid
RBM15	chromium hexavalent ion	IGF2BP2	Resveratrol
RBM15	decamethrin	IGF2BP2	sodium bichromate
RBM15	deoxynivalenol	IGF2BP2	Soot
RBM15	Estradiol	IGF2BP2	Succimer
RBM15	Ethanol	IGF2BP2	triptonide
RBM15	Fenthion	IGF2BP2	vinylidene chloride
RBM15	Folic Acid	RBM15	Choline
RBM15	hexabromocyclododecane	RBM15	Inulin
RBM15	Hydrogen Peroxide	RBM15	Methionine
RBM15	methidathion	RBM15	N-Methyl-3,4-methylenedioxyamphetamine
RBM15	monobutyl phthalate	RBM15	perfluorooctane sulfonic acid
RBM15	Nanotubes, Carbon	RBM15	pirinixic acid
RBM15	N-(oxo-5,6-dihydrophenanthrid	RBM15	Resveratrol

	in-2-yl)-N, N-dimethylacetamide hydrochloride		
RBM15	Tetrachlorodibenzodioxin	RBM15	Sodium Fluoride
RBM15	trimellitic anhydride	RBM15	triptonide
EIF3B	1,2-Dimethylhydrazine	RBM15	Vehicle Emissions
EIF3B	2,2',4,4'-tetrabromodiphenyl ether	WTAP	1,2-Dimethylhydrazine
EIF3B	Acetaminophen	WTAP	2,3',4,4',5-pentachlorobiphenyl
EIF3B	Carbon Tetrachloride	WTAP	Acetaminophen
EIF3B	chromium hexavalent ion	WTAP	Asbestos, Crocidolite
EIF3B	decamethrin	WTAP	casticin
EIF3B	deoxynivalenol	WTAP	Chlorodiphenyl (54% Chlorine)
EIF3B	Estradiol	WTAP	Clobetasol
EIF3B	Ethanol	WTAP	Cobalt
EIF3B	Fenthion	WTAP	decamethrin
EIF3B	Folic Acid	WTAP	Ethanol
EIF3B	hexabromocyclododecane	WTAP	ethylene dichloride
EIF3B	Hydrogen Peroxide	WTAP	Folic Acid
EIF3B	methidathion	WTAP	Miconazole
EIF3B	monobutyl phthalate	WTAP	Pentachlorophenol
EIF3B	Nanotubes, Carbon	WTAP	pirinixic acid
EIF3B	N-(oxo-5,6-dihydrophenanthridin-2-yl)-N,	WTAP	Tetrachlorodibenzodioxin

	N-dimethylacetamide hydrochloride		
EIF3B	Tetrachlorodibenzodioxin	WTAP	Valproic Acid
EIF3B	trimellitic anhydride	WTAP	Vehicle Emissions
IGF2BP2	1,2-Dimethylhydrazine	YTHDC1	1,2-Dimethylhydrazine
IGF2BP2	Acetaminophen	YTHDC1	Acetaminophen
IGF2BP2	Aflatoxin B1	YTHDC1	Aflatoxin B1
IGF2BP2	Benzo(a)pyrene	YTHDC1	Asbestos, Crocidolite
IGF2BP2	Carbon Tetrachloride	YTHDC1	Carbon Tetrachloride
IGF2BP2	Chloroprene	YTHDC1	chrysene
IGF2BP2	Choline	YTHDC1	Clobetasol
IGF2BP2	decamethrin	YTHDC1	Inulin
IGF2BP2	Diquat	YTHDC1	Nanotubes, Carbon
IGF2BP2	epoxiconazole	YTHDC1	Particulate Matter
IGF2BP2	Ethanol	YTHDC1	perfluorooctane sulfonic acid
IGF2BP2	Fenretinide	YTHDC1	Tetrachlorodibenzodioxin
IGF2BP2	Folic Acid	YTHDC1	titanium dioxide
IGF2BP2	folpet	YTHDC1	Troglitazone
IGF2BP2	Isoproterenol	YTHDC1	Tungsten
IGF2BP2	Magnetite Nanoparticles	YTHDC1	Vehicle Emissions
IGF2BP2	Methionine		

“mRNA”and“drung”represent node; “-”represent edge
