# Supplementary figures and images for: Integration of bioinformatics and identification of the role of m6A genes in NAFLD
Source: PLoS One. 2025 May 28;20(5):e0321757. doi: 10.1371/journal.pone.0321757 (PMC12119021; doi:10.1371/journal.pone.0321757)

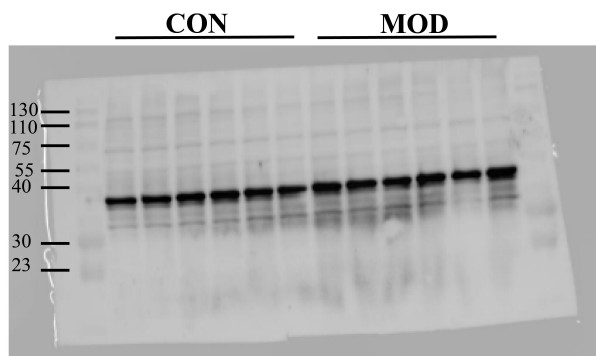

**GAPDH 36KD**

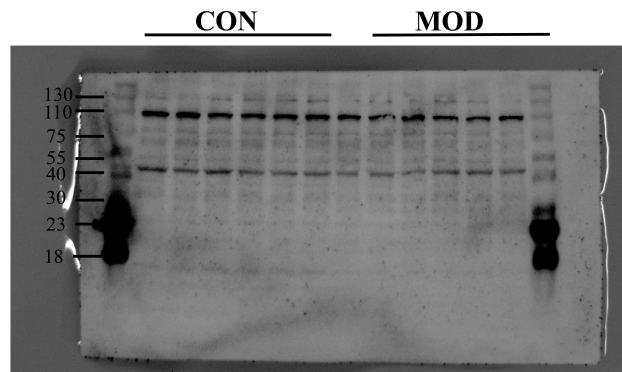

**EIF3B 93KD**

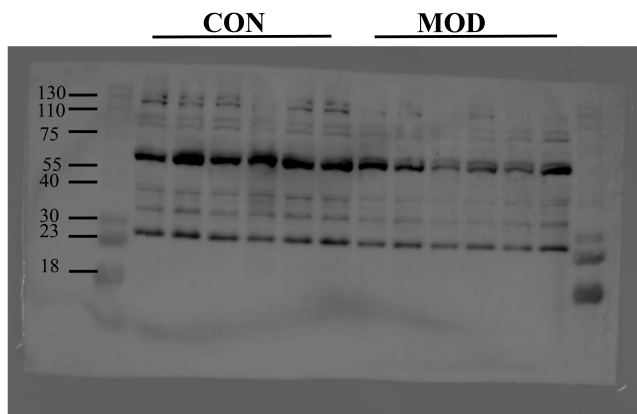

**IGF2BP2 65KD**

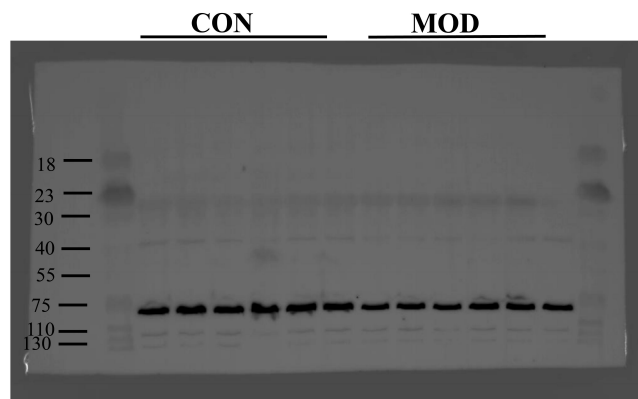

**YTHDC1 85KD**

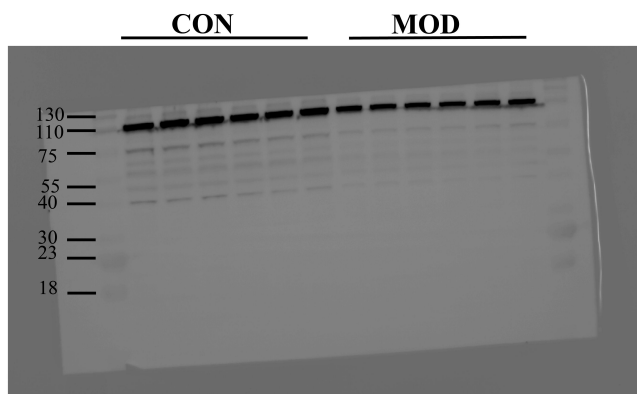

**RBM15 105KD**

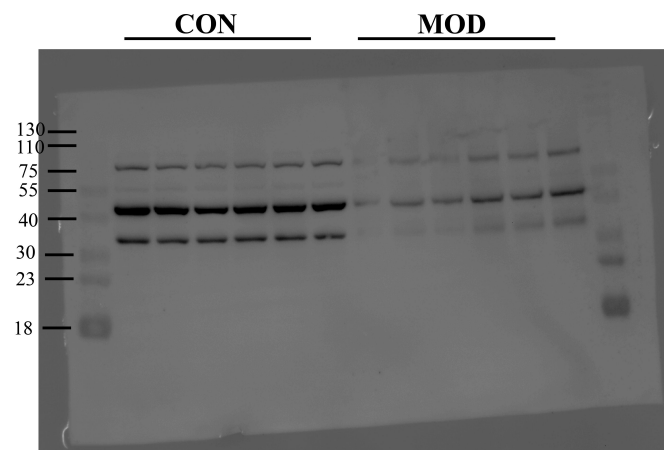

**WTAP 45KD**

Supplement: S9 Fig — (PDF) [file pone.0321757.s009.pdf]
