# Supplementary material for: Integration of bioinformatics and identification of the role of m6A genes in NAFLD
Source: PLoS One. 2025 May 28;20(5):e0321757. doi: 10.1371/journal.pone.0321757 (PMC12119021; doi:10.1371/journal.pone.0321757)
Supplement: Attachment — Submitted filename: Response to Reviewers.docx [file pone.0321757.s011.docx]

**Response Letter**

Manuscript number: PONE-D-25-03540

Titlie: Integration of bioinformatics and identification of the role of m6A genes in NAFLD

Dear Editors and Reviewers,

Thank you for your reminding. You and the other reviewers’ comments are all great importance to our article. All these comments have contributed a lot to improve the quality of our article. After this revision, we have written a point-by-point rebuttal/response letter to you as you can see above. Meanwhile, we also wrote a point-by-point response letter to the other nice reviewers to acknowledge their helps, In this response letter, the reviewers' comments are presented in italics, and our corresponding changes and additions to the manuscript are highlighted in red text. We have tried our best to make all the revisions clear, and we hope that the revised manuscript meets the requirements for publication.

PONE-D-25-03540

Integration of bioinformatics and identification of the role of m6A genes in NAFLD

PLOS ONE

Dear Dr. Qi,

Thank you for submitting your manuscript to PLOS ONE. After careful consideration, we feel that it has merit but does not fully meet PLOS ONE’s publication criteria as it currently stands. Therefore, we invite you to submit a revised version of the manuscript that addresses the points raised during the review process.

Please submit your revised manuscript by Apr 09 2025 11:59PM. If you will need more time than this to complete your revisions, please reply to this message or contact the journal office at [plosone@plos.org](mailto:plosone@plos.org). When you're ready to submit your revision, log on to https://www.editorialmanager.com/pone/ and select the 'Submissions Needing Revision' folder to locate your manuscript file.

Please include the following items when submitting your revised manuscript:

- A rebuttal letter that responds to each point raised by the academic editor and reviewer(s). You should upload this letter as a separate file labeled 'Response to Reviewers'.
- A marked-up copy of your manuscript that highlights changes made to the original version. You should upload this as a separate file labeled 'Revised Manuscript with Track Changes'.
- An unmarked version of your revised paper without tracked changes. You should upload this as a separate file labeled 'Manuscript'.

If you would like to make changes to your financial disclosure, please include your updated statement in your cover letter. Guidelines for resubmitting your figure files are available below the reviewer comments at the end of this letter.

If applicable, we recommend that you deposit your laboratory protocols in protocols.io to enhance the reproducibility of your results. Protocols.io assigns your protocol its own identifier (DOI) so that it can be cited independently in the future. For instructions see: [https://journals.plos.org/plosone/s/submission-guidelines#loc-laboratory-protocols](https://track.editorialmanager.com/CL0/https://journals.plos.org/plosone/s/submission-guidelines%23loc-laboratory-protocols/1/010f019535bde6ee-793b09f3-c42b-4e70-b26c-f69603019e07-000000/XGOOpuMQAJWH4ibBW_G6B5pFaKx7DCwZteewujKyL5Y=199). Additionally, PLOS ONE offers an option for publishing peer-reviewed Lab Protocol articles, which describe protocols hosted on protocols.io. Read more information on sharing protocols at [https://plos.org/protocols?utm_medium=editorial-email&utm_source=authorletters&utm_campaign=protocols](https://track.editorialmanager.com/CL0/https://plos.org/protocols?utm_medium=editorial-email&utm_source=authorletters&utm_campaign=protocols/1/010f019535bde6ee-793b09f3-c42b-4e70-b26c-f69603019e07-000000/gdUEVt90P0QGVKQMU72eGBc3a30SmKRXnEGbEdFX4nM=199).

We look forward to receiving your revised manuscript.

Kind regards,

Jinhui Liu
Academic Editor
PLOS ONE

Journal Requirements:

When submitting your revision, we need you to address these additional requirements.

1.Please ensure that your manuscript meets PLOS ONE's style requirements, including those for file naming. The PLOS ONE style templates can be found at 
https://journals.plos.org/plosone/s/file?id=wjVg/PLOSOne_formatting_sample_main_body.pdf and 
<https://journals.plos.org/plosone/s/file?id=ba62/PLOSOne_formatting_sample_title_authors_affiliations.pdf>

Comment 1: Thank you for reviewing my manuscript, I have carefully checked the article, checked the publication requirements of PLOS ONE journals, and confirmed that the format is correct.

2.To comply with PLOS ONE submissions requirements, in your Methods section, please provide additional information regarding the experiments involving animals and ensure you have included details on (1) methods of sacrifice, (2) methods of anesthesia and/or analgesia, and (3) efforts to alleviate suffering.

Comment 2: Thank you for reviewing my manuscript. To improve the transparency of the article, I have supplemented the full dose of the drug-pentobartal sodium used in line 217, page 10.

3.Thank you for stating the following financial disclosure: Study design: the National Natural Science Foundation of China (Grant number [82460794]) , Xingdian Talent Support Program-Youth Talent Special Project (2023. No. 166).
Data collection and analysis:High-level TCM talents: (reserve talents) project of Yunnan (2021. No. 1)
Please state what role the funders took in the study.  If the funders had no role, please state: ""The funders had no role in study design, data collection and analysis, decision to publish, or preparation of the manuscript."" 
If this statement is not correct you must amend it as needed. 
Please include this amended Role of Funder statement in your cover letter; we will change the online submission form on your behalf.

Comment 3: Thank you for reviewing my manuscript. The three projects mentioned in the article are all led by the corresponding author Yan Qi, and play the role of "project leader" in the three projects. The main role played by the funders was financial support and did not have additional roles in the study design, data collection and analysis, the decision to publish, or the preparation of the manuscript. I have written in my cover letter including this modification. Please change the online submission form on my behalf. We thank you again for your careful reading.

4.PLOS ONE now requires that authors provide the original uncropped and unadjusted images underlying all blot or gel results reported in a submission’s figures or Supporting Information files. This policy and the journal’s other requirements for blot/gel reporting and figure preparation are described in detail at https://journals.plos.org/plosone/s/figures#loc-blot-and-gel-reporting-requirements and https://journals.plos.org/plosone/s/figures#loc-preparing-figures-from-image-files. When you submit your revised manuscript, please ensure that your figures adhere fully to these guidelines and provide the original underlying images for all blot or gel data reported in your submission. See the following link for instructions on providing the original image data: https://journals.plos.org/plosone/s/figures#loc-original-images-for-blots-and-gels.   
In your cover letter, please note whether your blot/gel image data are in Supporting Information or posted at a public data repository, provide the repository URL if relevant, and provide specific details as to which raw blot/gel images, if any, are not available. Email us at [plosone@plos.org](mailto:plosone@plos.org) if you have any questions.

Comment 4: Thank you for reviewing my manuscript. As for the requirements of image data, I have made the whole film of gel imaging according to the guidelines of the journal. In addition, I will upload the whole original uncropped and unadjusted images as an attachment for the reviewers and editors to view. Please confirm that these measures meet the requirements of the journal. Thank you again for your patient review.

5. Please review your reference list to ensure that it is complete and correct. If you have cited papers that have been retracted, please include the rationale for doing so in the manuscript text, or remove these references and replace them with relevant current references. Any changes to the reference list should be mentioned in the rebuttal letter that accompanies your revised manuscript. If you need to cite a retracted article, indicate the article’s retracted status in the References list and also include a citation and full reference for the retraction notice.
   Comment 5: Thank you for reviewing my manuscript. We have verified all references against original sources to ensure formatting consistency (e.g., DOI, volume/issue, page numbers) and confirmed that all cited works are appropriately acknowledged., and that there are no retracted papers. Thanks again for your review.

Additional Editor Comments:

Authors should revise according to the suggestions of reviewers. The modifications should be marked. A point to point response letter is needed.


[Note: HTML markup is below. Please do not edit.]

Reviewers' comments:

Reviewer's Responses to Questions

**Comments to the Author**

1. Is the manuscript technically sound, and do the data support the conclusions?

The manuscript must describe a technically sound piece of scientific research with data that supports the conclusions. Experiments must have been conducted rigorously, with appropriate controls, replication, and sample sizes. The conclusions must be drawn appropriately based on the data presented.

Reviewer #1: Yes

2. Has the statistical analysis been performed appropriately and rigorously?

Reviewer #1: Yes

3. Have the authors made all data underlying the findings in their manuscript fully available?

The [PLOS Data policy](http://track.editorialmanager.com/CL0/http://www.plosone.org/static/policies.action%23sharing/1/010f019535bde6ee-793b09f3-c42b-4e70-b26c-f69603019e07-000000/yv6H0NU-SBD1Vp0GlE1dKzL8up0vH6wFOu4UVWfWKUY=199) requires authors to make all data underlying the findings described in their manuscript fully available without restriction, with rare exception (please refer to the Data Availability Statement in the manuscript PDF file). The data should be provided as part of the manuscript or its supporting information, or deposited to a public repository. For example, in addition to summary statistics, the data points behind means, medians and variance measures should be available. If there are restrictions on publicly sharing data—e.g. participant privacy or use of data from a third party—those must be specified.

Reviewer #1: Yes

4. Is the manuscript presented in an intelligible fashion and written in standard English?

PLOS ONE does not copyedit accepted manuscripts, so the language in submitted articles must be clear, correct, and unambiguous. Any typographical or grammatical errors should be corrected at revision, so please note any specific errors here.

Reviewer #1: Yes

5. Review Comments to the Author

Please use the space provided to explain your answers to the questions above. You may also include additional comments for the author, including concerns about dual publication, research ethics, or publication ethics. (Please upload your review as an attachment if it exceeds 20,000 characters)

Reviewer #1: *They empolyed gene sequencing and animal model to demonstrate the diagnostic model based on the five key genes. The work is systematic and comprehensive.The study provides valuable insights into the molecular mechanisms underlying NAFLD and highlights the potential of m6A RNA methylation as a therapeutic target for this disease. I am not the expert for bioinformation, there are some suggestions from molecular interaction view to potentially improve the manuscript.*

*1. The sample size and potential data bias should be considered, for example in Fig 5ABC.*

Comment 1: We appreciate the reviewer’s concerns regarding sample size and potential data bias. In this study, our analyses utilized a relatively robust sample cohort: the NAFLD dataset comprised 47 NAFLD cases and 31 controls, while the GSE135251 dataset included 206 NAFLD cases and 10 controls, collectively providing sufficient statistical power. To mitigate batch effects and technical variability across datasets, we implemented rigorous batch correction using the sva R package, ensuring data homogeneity prior to integrative analysis. Principal component analysis (PCA) results (Figs. 2C-D) demonstrated effective harmonization of expression patterns post-correction, confirming successful mitigation of inter-dataset batch effects.

Furthermore, cross-dataset validation was inherently incorporated through our multi-cohort integration strategy, substantially reducing bias inherent to single-dataset analyses. During feature selection and model development, we employed a dual-machine learning approach: initial identification of six candidate genes through support vector machine (SVM) optimization for maximal NAFLD diagnostic accuracy (Figs. 5A-B), followed by refinement via LASSO regression to establish a parsimonious five-gene signature (RBM15, IGF2BP2, EIF3B, YTHDC1, WTAP) (Figs. 5C-D). This sequential methodology enhances the biological plausibility and analytical robustness of our findings.

We will explicitly address these methodological considerations and potential limitations in the revised Discussion section (Page 32-33, Lines 587-610) to strengthen the contextual interpretation of our results.

We sincerely appreciate the reviewer’s insightful comments.

*2. The detailed molecular mechanisms by which the identified genes regulate m6A methylation might be explored through MD simulations in further studies.*

Comment 2: We sincerely appreciate the reviewer's constructive feedback on our work.This study employed a bioinformatics-driven approach to systematically identify and characterize NAFLD-associated m6A-related differentially expressed genes (m6A-DEGs). Through multi-tiered analytical strategies - including Gene Ontology (GO) enrichment profiling, machine learning framework development, and experimental validation - we elucidated the potential regulatory roles of these genes in NAFLD pathogenesis. Our findings provide novel insights into the molecular underpinnings of NAFLD, with core results rigorously validated through RT-qPCR and Western blot analyses.

Regarding molecular dynamics (MD) simulations, we fully acknowledge their critical value in deciphering protein-RNA interaction dynamics. However, the current methodological focus was strategically prioritized toward high-confidence identification and preliminary validation of m6A regulatory targets, coupled with technical constraints in computational resource allocation. While this phase did not incorporate MD-based mechanistic exploration, we are committed to integrating this powerful tool in subsequent investigations to resolve atomic-level details of m6A reader protein-RNA binding thermodynamics.

We have incorporated this methodological perspective into the revised manuscript (Page 32-33, Lines 587-610)

Thank you for your thorough evaluation and valuable suggestions to strengthen the mechanistic depth of our research.

6. PLOS authors have the option to publish the peer review history of their article ([what does this mean?](https://track.editorialmanager.com/CL0/https://journals.plos.org/plosone/s/editorial-and-peer-review-process%23loc-peer-review-history/1/010f019535bde6ee-793b09f3-c42b-4e70-b26c-f69603019e07-000000/5wYsYcVceTG4ZTeEq3KmxL_pxYUaoCXTOFfsYivs2gM=199)). If published, this will include your full peer review and any attached files.


If you choose “no”, your identity will remain anonymous but your review may still be made public.


**Do you want your identity to be public for this peer review?** For information about this choice, including consent withdrawal, please see our [Privacy Policy](https://track.editorialmanager.com/CL0/https://www.plos.org/privacy-policy/1/010f019535bde6ee-793b09f3-c42b-4e70-b26c-f69603019e07-000000/yDttz9N2MvKPS4_W9PFDgCDrwcv2NA3d5UEzz4cQASQ=199).

Reviewer #1: **Yes:** Jiebo Li

[NOTE: If reviewer comments were submitted as an attachment file, they will be attached to this email and accessible via the submission site. Please log into your account, locate the manuscript record, and check for the action link "View Attachments". If this link does not appear, there are no attachment files.]

While revising your submission, please upload your figure files to the Preflight Analysis and Conversion Engine (PACE) digital diagnostic tool, [https://pacev2.apexcovantage.com/](https://track.editorialmanager.com/CL0/https://pacev2.apexcovantage.com//1/010f019535bde6ee-793b09f3-c42b-4e70-b26c-f69603019e07-000000/VG-qVUSVGHqwqwT_iJuyZkU7Eo1M6jfbrL5T08bCAfY=199). PACE helps ensure that figures meet PLOS requirements. To use PACE, you must first register as a user. Registration is free. Then, login and navigate to the UPLOAD tab, where you will find detailed instructions on how to use the tool. If you encounter any issues or have any questions when using PACE, please email PLOS at [figures@plos.org](mailto:figures@plos.org). Please note that Supporting Information files do not need this step.

In compliance with data protection regulations, you may request that we remove your personal registration details at any time. [(Remove my information/details)](https://track.editorialmanager.com/CL0/https://www.editorialmanager.com/pone/login.asp?a=r/1/010f019535bde6ee-793b09f3-c42b-4e70-b26c-f69603019e07-000000/ahH2Dlq2gfcnBkRJ41RNEd3c5u3SKCAX0Z-kRyaLPtg=199). Please contact the publication office if you have any questions.

We tried our best to improve the manuscript and made some changes marked in red in revised paper which will not influence the content and framework of the paper. We appreciate for Editors/Reviewers' warm work earnestly, and hope the correction will meet with approval.

Best regards!

Yan Qi

The First Affiliated Hospital of Yunnan University of Chinese Medicine

China

[+86-15808702160]

qiyankm@163.com
